# *Mycobacterium tuberculosis* host cell interaction: Role of latency associated protein Acr-1 in differential modulation of macrophages

**DOI:** 10.1371/journal.pone.0206459

**Published:** 2018-11-05

**Authors:** Nida Mubin, Susanta Pahari, Mohammad Owais, Swaleha Zubair

**Affiliations:** 1 Molecular Immunology Laboratory, Interdisciplinary Biotechnology Unit, Aligarh Muslim University, Aligarh, India; 2 Immunology Laboratory, CSIR-Institute of Microbial Technology, Chandigarh, India; 3 Department of Computer Science, Aligarh Muslim University, Aligarh, India; Karolinska Institutet, SWEDEN

## Abstract

*Mycobacterium tuberculosis* (*M*.*tb*) contrives intracellular abode as a strategy to combat antibody onslaught. Additionally, to thrive against hostile ambiance inside host macrophages, the pathogen inhibits phago-lysosomal fusion. Finally, to further defy host cell offensives, *M*.*tb* opts for dormant phase, where it turns off or slows down most of its metabolic process as an added stratagem. While *M*.*tb* restrains most of its metabolic activities during dormancy, surprisingly latency-associated alpha-crystallin protein (Acr-1) is expressed most prominently during this phase. Interestingly, several previous studies described the potential of Acr-1 to induce the robust immuno-prophylactic response in the immunized host. It is intriguing to comprehend the apparent discrepancy that the microbe *M*.*tb* overexpresses a protein that has the potential to prime host immune system against the pathogen itself. Keeping this apparent ambiguity into consideration, it is imperative to unravel intricacies involved in the exploitation of Acr-1 by *M*.*tb* during its interaction with host immune cells. The present study suggests that Acr-1 exhibits diverse role in the maturation of macrophages (MΦs) and related immunological responses. The early encounter of bone marrow derived immune cells (pre-exposure during differentiation to MΦs) with Acr-1 (AcrMΦpre), results in hampering of their function. The pre-exposure of naïve MΦs with Acr-1 induces the expression of TIM-3 and IL-10. In contrast, exposure of fully differentiated MΦs to Acr-1 results in their down-modulation and induces the phosphorylation of STAT-1 and STAT-4 in host MΦs. Furthermore, Acr-1 mediated activation of MΦs results in the induction of Th1 and Th17 phenotype by activated T lymphocyte.

## Introduction

*Mycobacterium sps*. adapt intracellular niche as a stratagem to evade antibody onslaught. Incidentally, it chose macrophages, the first line defender of the host, as a sheltered resort [1, 2]. To withstand hostile ambiance inside the macrophages, the pathogen downplays its own metabolic activities and switches to a non-replicating dormant state [3, 4]. As suppression of invading *M*.*tb* is generally conferred by cell-mediated components of the host, residual latent bacilli residing inside macrophage remain viable in the healthy host for many years and can reactivate into contagious TB disease in subsequent years [5]. During latency, *M*.*tb* uses a range of effector modalities to modulate various host-related metabolic processes and factors *viz*. pattern recognition, antigen presentation, and phagolysosome formation *etc*. The incurred modulations help in the intracellular survival of *M*.*tb* inside host MΦs [6]. It is of paramount importance to comprehend the intricate host–pathogen interaction and the evasion approaches thrived by *M*.*tb* to circumvent immune onslaught of the host [2, 7]. The pathogen systematically deteriorates the immune function of the host by down-modulating overall activities and functioning of both macrophage and DC cell population [8]. While residing in a granuloma, *M*.*tb* expresses small molecular weight proteins i.e heat shock protein X (sHSPX) also known as HSP-16.3 or Acr-1. While the functions of various expressed protein including CFP-10, ESAT-6 i.e early secretory antigenic target of *M*.*tb*, 38-kDa, and 85B (Ag85B) had been evaluated for their possible role in modulating DCs [9], however to the best of our knowledge the effect of Acr-1 on host MΦs has remained elusive.

The protein Acr-1 is mainly expressed in the latent phase of *Mtb* infection and augmented in the stress condition [10]. The fact that *M*.*tb* devoid of Acr-1 fails to continue its latency [11], suggesting an imperious aspect of the protein in the prevailing of *M*.*tb* in the dormant state. With the present state of the knowledge, it is unclear that how *M*.*tb* exploits its latency associated antigen in modulating macrophage in terms of their maturation, differentiation, cytokine release and capacity to activate T cells. Although cellular immune responses help in the containment of *M*.*tb* infection, however, its persistence in MΦs ensues in down-regulation of costimulatory molecule on one hand and up-regulation of coinhibitory molecule on the other [12].

The infected MΦs may also incur tolerance and promotes *M*.*tb* survival [13]. Accumulating shreds of evidence have indicated that bone marrow derived, antigen-presenting cells (APCs) play a decisive role in the induction of T cell tolerance. The phenotype of tolerance depends on antigen presenting efficiency of the specific class of APCs [14]. An increase in regulatory T cells (Tregs) population has also been noticed in latent TB which eventually restrains type 17 T-helper (Th17) cells function. The overall, development in Treg populations along with the decline in Th17 cells generally leads to suppression of the immune response against *M*.*tb* [15].

In general, antigen presenting cells including MΦs participate in a pivotal role in suppressing immunity against *M*.*tb* and facilitate differentiation of naïve T cells to effector cells [2, 7, 16, 17]. The invading pathogen in turn modulates host immune machinery that leads to induction of tolerance that otherwise eventually help its safe survival inside the host [7]. For example, latent *M*.*tb* residing in macrophages exploits host cell machinery to inhibit the function of various immune cells. It also manipulates the differentiation of monocytes and macrophages [18, 19].

Several sets of studies had suggested the immunoprophylactic role of Acr-1 in the immunized host [20]. Macrophages harnessing to various tuberculosis antigen may provide a pivotal regulatory factor in endowing defensive immunity. Currently available studies have suggested that several *M*.*tb* proteins can modulate MΦs in various ways. For example, mycobacterial proteins such as ESX, ESAT-6, and CFP-10 had been stated to facilitate maturation of MΦs [21–23]. The expressed mycobacterial proteins also prime T cell response by triggering NF-κB and MAPK signaling pathways and urge Th-1 immune response in the host, most presumably by augmenting IL-12 and IFN-γ production [24, 25].

The present study explores a comprehensive approach to study the immunological role of Acr-1 in the modulation of the host MΦs. The data suggest that Acr-1 hamper the maturation and differentiation of bone marrow derived macrophages (BMDM). The BMDM exhibited tolerogenic phenotype with the hampered immune response when exposed to Acr-1 during their maturation by modulating STAT-1 and STAT-4 pathways.

## Material and method

### Mice

BALB/c and C57BL/6 mice aged 6–8 wk old were obtained from the Department animal house facility Interdisciplinary Biotechnology Unit, AMU Aligarh and JALMA Institute for Leprosy and other Mycobacterial Diseases, Agra, India. Animals were offered standard pellet diet and water *ad libitum*. The animals were maintained in pathogen-free conditions at the Department Animal House Facility.

### Ethics statement

The animal experiments were approved by the Institutional Animal Ethics Committee of the Interdisciplinary Biotechnology Unit, Aligarh Muslim University, Aligarh, India. All animal experiments were performed according to the National Regulatory Guidelines issued by the CPCSEA (Committee for the Purpose of Control and Supervision of Experiments on Animals). Our approval ID was 332/ CPCSEA, Ministry of Environment and Forests, Paryavaran Bhavan, Government of India.

### Reagents

Both free and fluorochrome conjugated antibodies (Abs): CD4 Pacific Blue, CD-80-FITC, CD-40-PEcy5, CD86-PE, MHC-II-Per-CPcy5-5, TIM-3-APC. All reagents used in cytokine ELISA experiment were procured from eBiosciences (San Diego, CA) and BD Pharamigen (San Diego, CA). Fetal Bovine Serum (FBS) was purchased from GIBCO (Grand Island, NY) and Biological Industries (Kibbutz Beit Haemek 25115, Israel). Penicillin, Streptomycin, L-glutamine, L-pyruvate and tissue culture grade plastic wares used in experiments were purchased from Serva (Heidelbergh, Germany) and BD Biosciences (Bedford, MA). Abs against pSTAT-1 and pSTAT-4 were procured from BD Biosciences (San Diego, CA). All other reagents and chemicals were purchased from Sigma Aldrich (St.Louis, MO) or otherwise mentioned.

### Murine bone marrow derived macrophages (BMDM)

Bone marrow cells were collected from femurs and tibiae of C57BL/6 mice. Briefly, a single suspension of bone marrow cells (BMCs) collected from the femurs and tibia of C57BL/6 mice was obtained by passing the bone marrow spicules through cell strainers [26]. Cells were counted (2x10^6^/well) and cultured with RPMI medium supplemented with 20% L929 media contains M-CSF with 10% FBS in tissue culture plates in presence of Acr-1 [9μg/ml] (AcrMΦpre). On day 3, the cells were replenished with fresh media containing Acr-1 in the first set of experiment (AcrMΦpre). In another set, BMDM cells were cultured in similar condition in media without Acr-1. On sixth day, the cells of second set were exposed to Acr-1 [9μg/ml] (AcrMΦpost). On day 7, AcrMΦpre and AcrMΦpost cells were harvested and the supernatant was collected to determine expressed cytokines.

### Differentiation of THP-1 macrophages

Human THP-1 monocytes (3×10^5^/well) were incubated with PMA (20 ng/ml) in the presence or absence of Acr-1 (9μg/ml) for 16h in 48 well plates. The MΦs were washed with RPMI and then rested for another 24h. For the post-exposure to Acr-1 (AcrMΦpost) after differentiation, human macrophages were treated with Acr-1 (9 μg/ml) for 24h.

### Antigen uptake studies

For antigen uptake study, both AcrMΦpre and AcrMΦpost cells were incubated with fluorescein-isothiocyanate-dextran (100μg/ml) at 37°C for 30 mins. The cells incubated at 4°C were used as a negative control. Antigen uptake was detained by adding ice-cold phosphate buffered saline (PBS) at 4°C. Further, cells were washed subsequently 3 times with chilled PBS with 1% fetal bovine serum. Later, cells were acquired for the fluorescence-thiocyanate dextran (Dextran–FITC) using FACS Calibur and analyzed by BD DIVA (BD Biosciences, San Jose, CA) software.

### T cell proliferation study

CD4 specific T cells purified from splenocytes of BALB/c mice by MACS were labelled with CFSE-dye. The experimental AcrMΦpre and AcrMΦpost cells (cultured from C57BL/6 mice bone marrow) were irradiated at 3000 rad followed by co-culture with allogenic CD4 T cells in a 1:10 ratio (MΦs:T cells) for 72 hours. Then, cells were harvested, washed with 1X PBS and fixed in 1% paraformaldehyde. Flowcytometry data were acquired using FACS Aria II and analyzed by FACS DIVA software (BD Biosciences, San Jose, CA). Flowcytometry data is represented as percentage population normalized with suitable isotype-matched controls. Later, supernatant was collected to screen for the presence of various cytokine.

### Flow cytometry analysis

The cultured cells were stained with fluorochrome labelled anti-mouse surface Abs along with relevant isotype controls, as mentioned earlier study [27]. Briefly, cells were stained with fluorochrome-labelled anti-mouse F4/80/CD80/CD86/CD40/MHC-II/TIM-3 Abs and their isotype-matched controls. Finally, cells were washed and fixed in 1% paraformaldehyde. Flowcytometry data were obtained using FACS Aria II and analyzed by FACS DIVA software (BD Biosciences, San Jose, CA). Flowcytometry data is presented as percentage cells population positive for respective fluorochrome-labelled anti-mouse Abs normalized with suitable isotype-matched controls.

### Cytokines estimation

The culture supernatants from the various experimental set were assayed for the presence of cytokines such as IL-6, IL-12, TNF-α, IL-10, IFN-γ, and IL-17 as described previously [28]. In brief, 96 well ELISA plates were coated with purified rat anti-mouse IL-6, IL-12, TNF-α, IL-10, IFN-γ, and IL-17 Abs at 4°C overnight. Then, the plates were washed 3 times with FACS buffer (1X PBS+0.05% Tween-20) and blocking was performed with blocking buffer (1X PBS+1% BSA) for 2h at RT and washed again 3 times with 1X PBS. Later, the culture supernatants and serially diluted standards (recombinant anti-mouse IL-6, IL-12, TNF-α, IL-10, IFN-γ, and IL-17 in log2 dilutions) were added in those plates and incubated for 2h. Then, the plates were again washed and bound IL-6, IL-12, TNF-α, IL-10, IFN-γ, and IL-17 antibody were detected by biotinylated anti-mouse secondary Abs, followed by their detection with avidin-HRP/OPD-H_2_O_2_ for colorimetric cytokines estimation. Later on, the concentrations of released cytokines were estimated using optical densities (ODs) of respective standard values as reference curves and data were presented as pg/ml.

### Annexin and Propidium Iodide (PI) assay

The Annexin V and PI assay were set according to the standard protocol, as stated previously [29]. In brief, both pre and post Acr-1 treated BMDM cells were harvested. After extensive washing step cells were resuspended in binding buffer solution [0.01 M HEPES (pH 7.4), 0.14 M NaCl, and 2.5 mM CaCl_2_]. The FITC conjugated Annexin V antibody was given to the cells. Further, the cells were incubated in dark for 15 mins at RT. Later, the cells were incubated with PI for 5 mins at RT and then the binding buffer (300 μl) was added. The stained samples were acquired immediately using FACS Aria II and data was analyzed by FACS DIVA software (BD Biosciences, San Jose, CA).

### Western blot

The immune cells belonging to both AcrMΦpre and AcrMΦpost groups were harvested and treated with lysis buffer (Cytosolic protein extraction buffer along with protease as well as phosphatase inhibitor cocktail). Equal amount of lysate was subjected to SDS-PAGE analysis. The gel was transblotted to PVDF membrane followed by subsequent blocking. The membrane was immunoblotted with specific Abs against phosphorylated STAT-1, and STAT-4. The blot was developed by a chemiluminescence kit (Amersham Pharmacia Biotech, Buckinghamshire, UK). The scanning of the blots was completed with the help of ImageQuant LAS 4000 (GE Healthcare, Pittsburgh, PA, USA). Densitometric analysis was done using ImageJ software.

### Statistical analysis

The data was analyzed by Student’s ‘t’ test, non-parametric Mann-Whitney two-tailed and repeated measure ANOVA with post-Student-Newman-Keuls multiple comparison test using Graph Pad Prism 6 software. The statistical differences were considered significant at a level of p <0.05.

## Result

### *M*.*tb* Acr-1 modulates the differentiation of host MΦs

Latent stage of *M*.*tb* infection is considered to be the foremost burden in developing countries. While residing inside MΦs, *M*.*tb* thwarts hostile host immune response by its transition to latent phase [30]. The infected MΦs undergo subtle changes upon establishment of infection. Macrophages express several surface molecules that actively participate in various cell-cell interaction events. Among various surface molecule, optimum expression of MHC as well as costimulatory molecules on APCs is imperative for deciding its future interaction with effector T cells in term of their activation or anergy [31].

**Fig 1:**
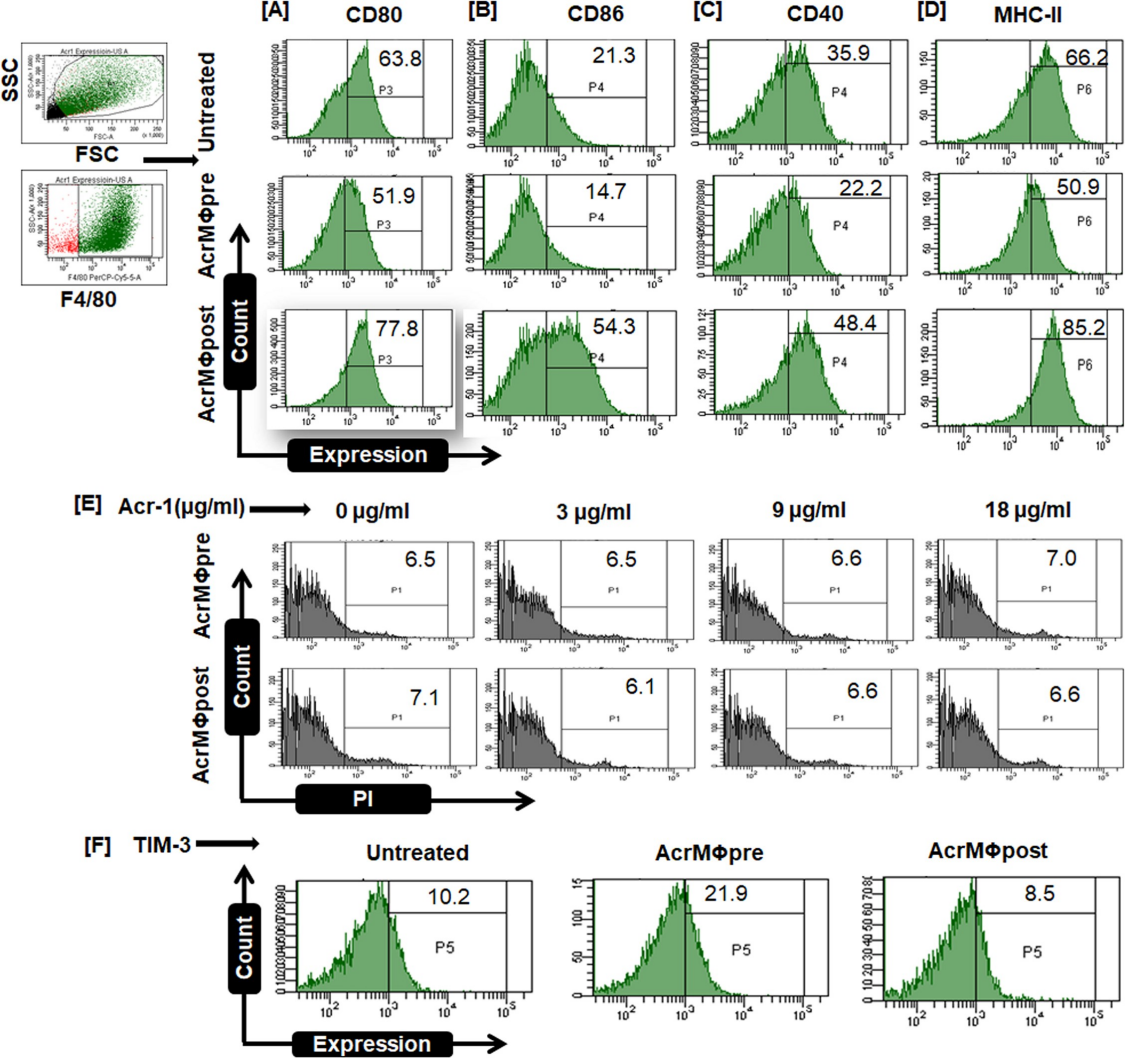
Differential role of alpha crystallin protein (Acr-1) in BMDM maturation. The BMDM were exposed to Acr-1 (9μg/ml) either during maturation (AcrMΦpre) or after maturation (AcrMΦpost) and the expression of various molecules on F4/80 gated population was assessed by flow cytometry. **[****A]**. The expression of CD80, **[B]** CD86, **[C ]** CD40, **[D]** MHC-II and **[E]** the cell viability was detected by PI staining **[F]** TIM-3 were monitored by flow cytometry. The value in the flow cytometry histogram depicts percentage of cells. Data are representative of 2–3 independent experiments.

In the present study, we envisage effect of *M*.*tb* latent phase expressed protein Acr-1 on modulation of MΦ functions. We noticed that presence of Acr-1 during differentiation and maturation phase of MΦs (AcrMΦpre) down-regulate the expression of CD80 molecules in the exposed MΦs. In contrast, exposures of mature MΦs to Acr-1 (AcrMΦpost) result in up-regulated expression of CD80 **(Fig 1A).** The AcrMΦpre modulated expression of co-stimulatory molecules resulted in downregulation of CD86, CD40, and MHCII molecules **(Fig 1B–1D).** In addition, Acr-1 also modulates the expression of costimulatory molecules in human monocyte differentiated macrophages. Interestingly, we observed upregulation of CD40 molecules upon exposure of mature human macrophages to Acr-1 (AcrMΦpost). On the contrary, exposure with Acr-1 (AcrMΦpre) during the differentiation of human macrophages resulted in the down regulation in the expression of CD40 **(S1A and S1B Fig)**. In order to validate the specificity of our findings, we have employed another *M*.*tb* antigen (CFP-10) and observed no considerable effect in both AcrMΦpre and AcrMΦpost MΦs **(S1A and S1B Fig)**. To authenticate that the downregulation of costimulatory molecules in AcrMΦpre is not due to cell death, we stained AcrMΦpre and AcrMΦpost (dose dependent, with varying dose of Acr-1) with PI and found no change in the viability of both AcrMΦpost and AcrMΦpre. The presence of Acr-1 did not affect the viability of host MΦ cells **(Fig 1E).**

### Exposure of MΦs to Acr-1 during their maturation results in the tolerogenic phenotype

Macrophages are the first line of host defense and actively participate in T cell activation. The invading *M*.*tb* finds the safe and protected niche inside the MΦs [32]. *M*.*tb* down modulates an immune function of host MΦs. Among various anergy inducing factor the higher expression of TIM-3 does hamper cytokine expression and leads to immune tolerance [33]. Keeping this fact into contemplation, we assessed the effect of Acr-1 on the expression of TIM-3 molecule. Acr-1 exposure induced a significantly higher the expression of TIM-3 in MΦs belonging to AcrMΦpre **(Fig 1F)**. In contrast, we observed lower expression of TIM-3 in AcrMΦpost macrophages **(Fig 1F)**.

### Acr-1 treatment modulates cytokine release in the treated macrophages

**Fig 2:**
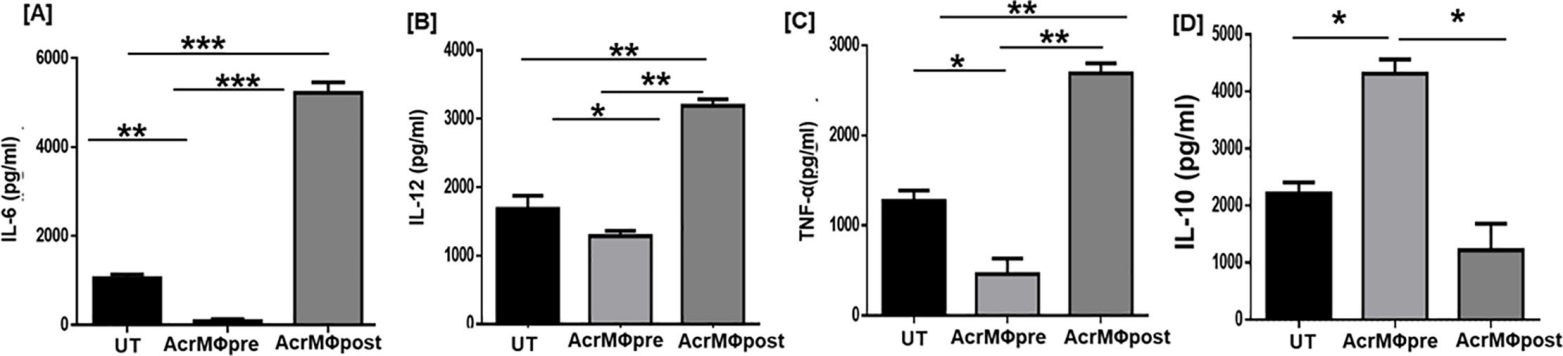
Acr-1 modulates the release of various cytokines. BMDM were generated in the presence of Acr-1 (9μg/ml) during pre-and post-maturation (AcrMΦpre and AcrMΦpost) and supernatant was collected and analyzed for the presence of **[A]** IL-6; **[B]** IL-12; **[C]** TNF-α; **[D]** IL-10 cytokines by ELISA. UT: untreated BMDM; AcrMΦpre: Acr-1 treatment during differentiation of BMDM; AcrMΦpost: Acr-1 treatment after differentiation of BMDM. Data represents as pg/ml (mean±SEM) and are representative of 3 independent experiments. Data were analyzed by one-way ANOVA repeated measure *p<0.05, **p<0.01, ***p<0.001.

The cytokines expressed by MΦs have been reported to play a significant role in differentiation of T cells [34]. IL-12 is a key cytokine that promotes differentiation of naïve T cells to Th1 phenotype to produce IFN-γ by T cells. The pro-inflammatory cytokine such as TNF-α, IL-6 etc. exerts primordial role in restricting the growth of *M*.*tb* [35]. On the other hand, IL-10 promotes pathogen persistence by inducing to *M*.*tb* phagosome maturation arrest in macrophages [36]. Considering this fact, we examined the potential of Acr-1 to induce IL-6, IL-12, TNF-α, and IL-10 cytokines secretion in both pre and post treatment groups **(Fig 2A–2D)**. We observed significant increase in the production of IL-6 (p<0.001), IL-12 (*p*<0.01), and TNF-α (p<0.01) in AcrMΦpost treatment **(Fig 2A–2C)**. Co-incubation of BMDM with Acr-1 during its maturation (AcrMΦpre) resulted in down-regulation of TNF-α. On the contrary, post exposure with Acr-1 resulted in significantly enhanced expression of TNF-α by BMDM **(Fig 2C)**. Further, there was significantly (*p*<0.05) higher expansion of IL-10 in AcrMΦpre group when compared to AcrMΦpost group **(Fig 2D)**. The overall data signify that Acr-1 incapacitate the maturation and expression of the key surface molecules that are potential for the optimum activation of MΦs.

### Acr-1 post treatment augments antigen uptake of treated macrophages

**Fig 3:**
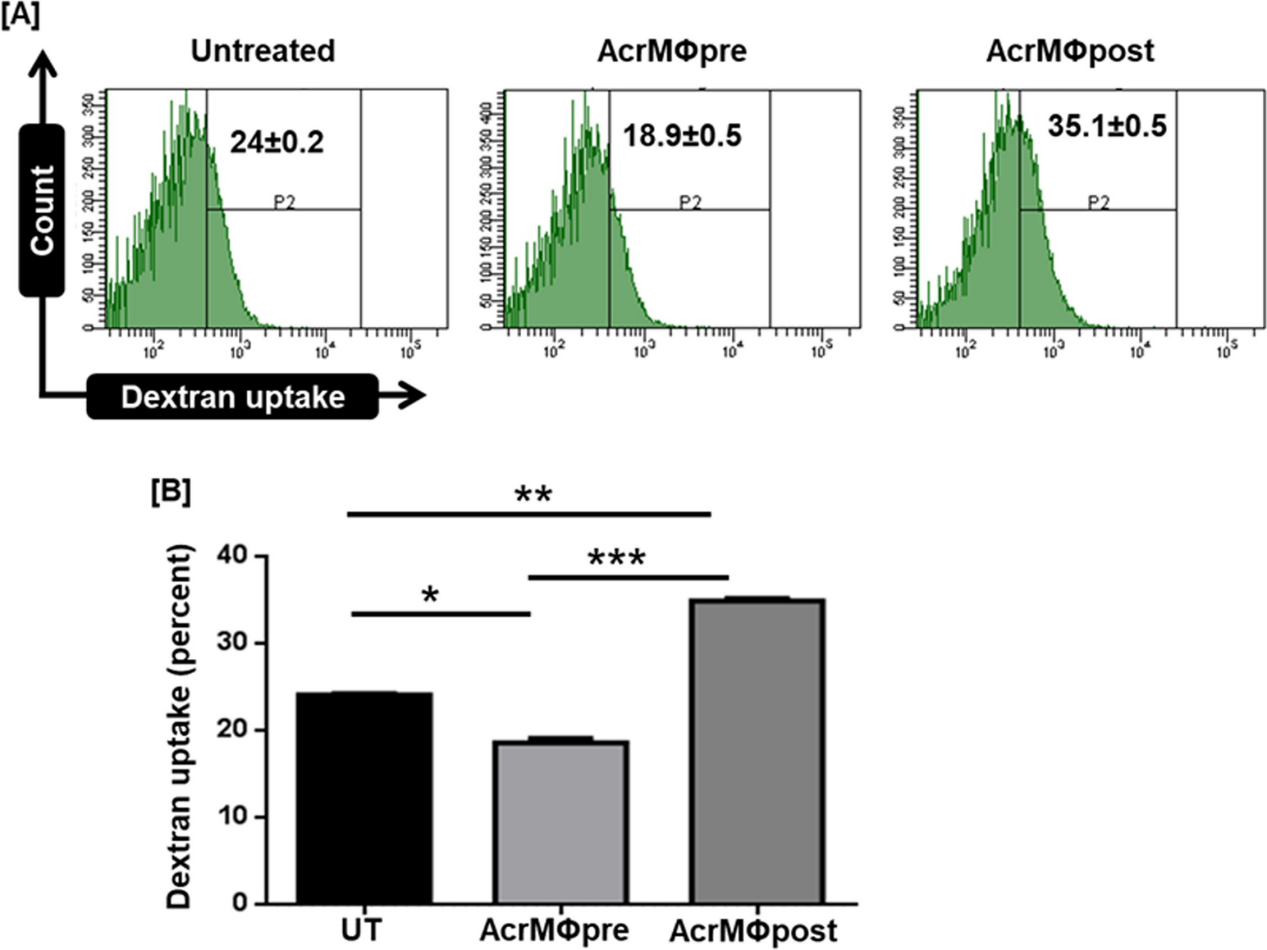
Acr-1regulates antigen uptake by macrophages. AcrMΦpre and AcrMΦpost were cultured in the existence of indicated dose of Acr-1. Antigen uptake was observed as **[A]** histogram; **[B]** bar graph depicts dextran uptake capacity of AcrMΦpre and AcrMΦpost macrophages by flow cytometry. Data represents mean±SEM and are representative of 2 independent experiments. Data were analyzed by one-way ANOVA repeated measure *p<0.05, **p<0.01, ***p<0.001.

AcrMΦpost treatment resulted in the up-regulation of costimulatory on one hand and down-regulation of coinhibitory molecule (CD80^hi^/CD86^hi^/CD40^hi^/MHC-II^hi^/TIM-3^lo^) in the expressed MΦs **(Fig 1B–1F).** We next assessed antigen uptake potential of MΦs in both AcrMΦpre and AcrMΦpost groups. The flowcytometry data exhibited a significant increase (p<0.01) in the dextran uptake of AcrMΦpost as compared to unstimulated control **(Fig 3A and 3B)**. AcrMΦpre treatment significantly (p<0.001) reduced dextran uptake capacity as compared to AcrMΦpost macrophages **(Fig 3A and 3B)**.

### AcrMΦpost treatment resulted in augmented proliferation of allogenic T cells while AcrMΦpre inhibits T cell proliferation

**Fig 4:**
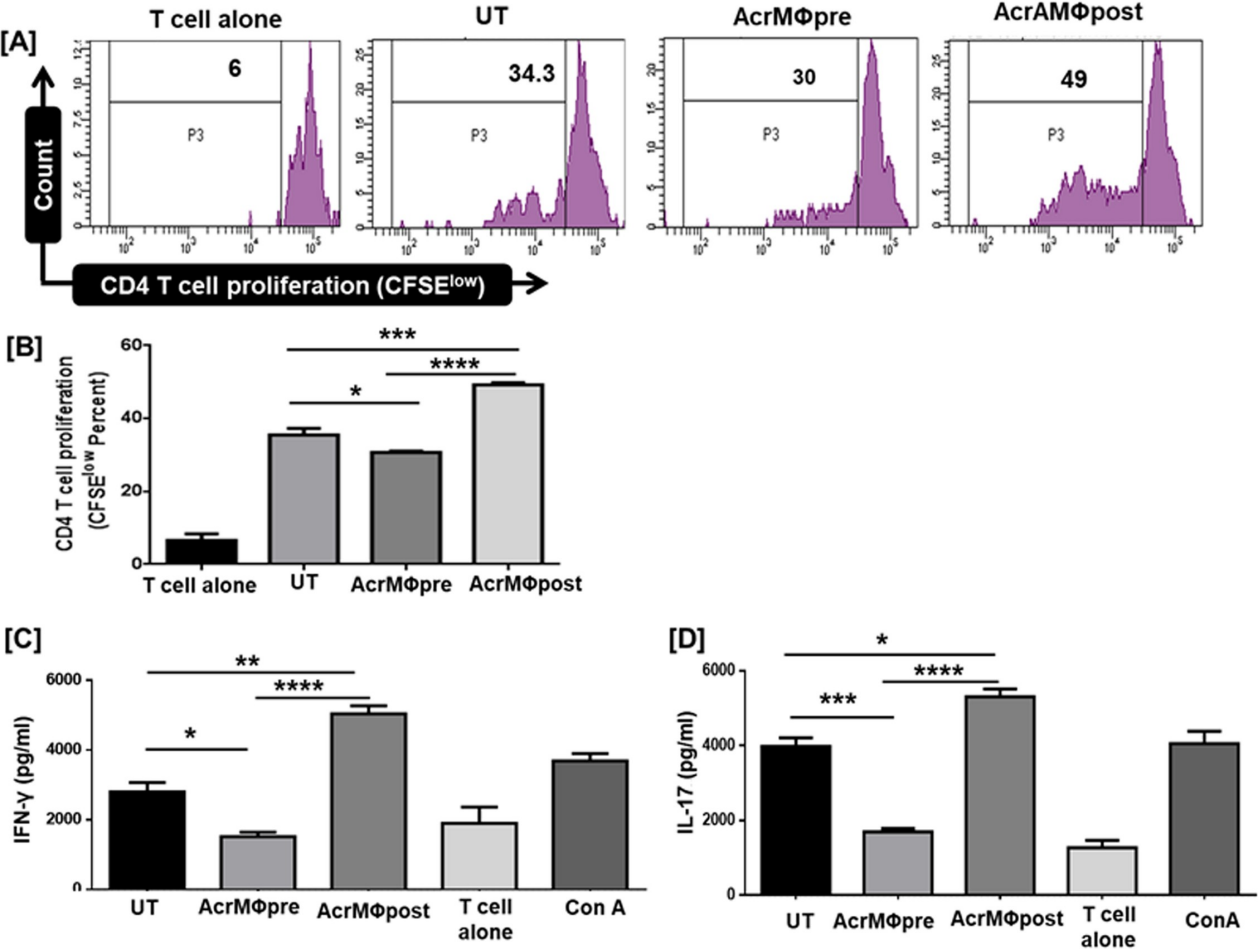
Post Acr-1 treatment resulted in augmented proliferation of allogenic T cell while pre Acr-1 treatment hampers T cell response. CD4 T cells purified by MACS were labelled with CFSE-dye. Later, BMDM primed with 9μg/ml Acr-1 (AcrMΦpre and AcrMΦpost) were co-cultured with allogenic CD4 T cells for 72h. **[A, B]** The CD4 T cell proliferation was examined by flowcytometry; **[B]** supernatant was collected for the estimation of IFN-γ; **[C]** IL-17 by ELISA. AcrMΦpre suppress, in contrast AcrMΦpost promotes the release of IFN-γ and IL-17 when co-cultured with allogenic (Balb/c) naïve T cell. The data signify as pg/ml (mean±SEM) and are representative of 3 independent experiments. Data were analyzed by one-way ANOVA repeated measure *p<0.05, **p<0.01, ***p<0.001, ****p<0.0001.

Macrophages play a pivotal role in the activation as well as differentiation of naïve CD4 T cells [2, 7]. Next, we explored the ability of AcrMΦpre and AcrMΦpost treated macrophages to activate T cells. Both AcrMΦpre and AcrMΦpost treated sets of macrophages were co-cultured with allogenic purified CD4 T cells. Interestingly, we observed that AcrMΦpost treated macrophages acquired the capacity to augment the T cells proliferation significantly (*p*<0.001) as compared to AcrMΦpre treated macrophages **(Fig 4A and 4B)**.

We noticed diverse role of AcrMΦpre and AcrMΦpost in modulating the release of IL-6 and IL-12 that play a pivotal role in the differentiation of T lymphocytes with Th1 and Th17 phenotype **(Fig 2A and 2B)**. Further, we assessed the influence of AcrMΦpre and AcrMΦpost treated macrophages in the production of Th1 and Th17 cells. Intriguingly, while AcrMΦpost potentiated however, AcrMΦpre treatment inhibited the differentiation of Th1 cells, as evidenced by elevated expression of IFN-γ by AcrMΦpost. The observed change was observed for both IFN-γ and Th-17 cytokines in allogeneic CD4 T cells **(Fig 4C and 4D).**

### Acr-1 modulates the phosphorylation of STAT-1 and STAT-4 in host MΦs

Besides playing a central role in evoking innate immune responses, macrophages are considered to be equally important in the induction of adaptive responses in the host [2, 7]. Various cytokines induced in response to antigen, initiates signaling cascade through there receptor, leading to the activation of the transcription factors that in turn target gene expression and activate the STATs, the cytoplasmic transcription factors. Binding with cytokines ensues in auto phosphorylation of receptor-associated JAK kinases that in turn phosphorylate and activate STATs. Activated STATs translocate into the nucleus and bind to specific target elements of the promoters of cytokine-inducible genes. Upon interaction with *M*.*tb* antigen, macrophages secrete various cytokines. Both STAT-1 and STAT4 can be activated by *M*.*tb* infected macrophages. IL-12 signaling activates STAT-4 that ensues in differentiation of naïve T cells into Th1 cells. On the other hand, IFN-γ executes multiple biological functions through the activation of STAT-1, a decisive transcription factor accountable for the activation as well as maturation of MΦs [37].

**Fig 5:**
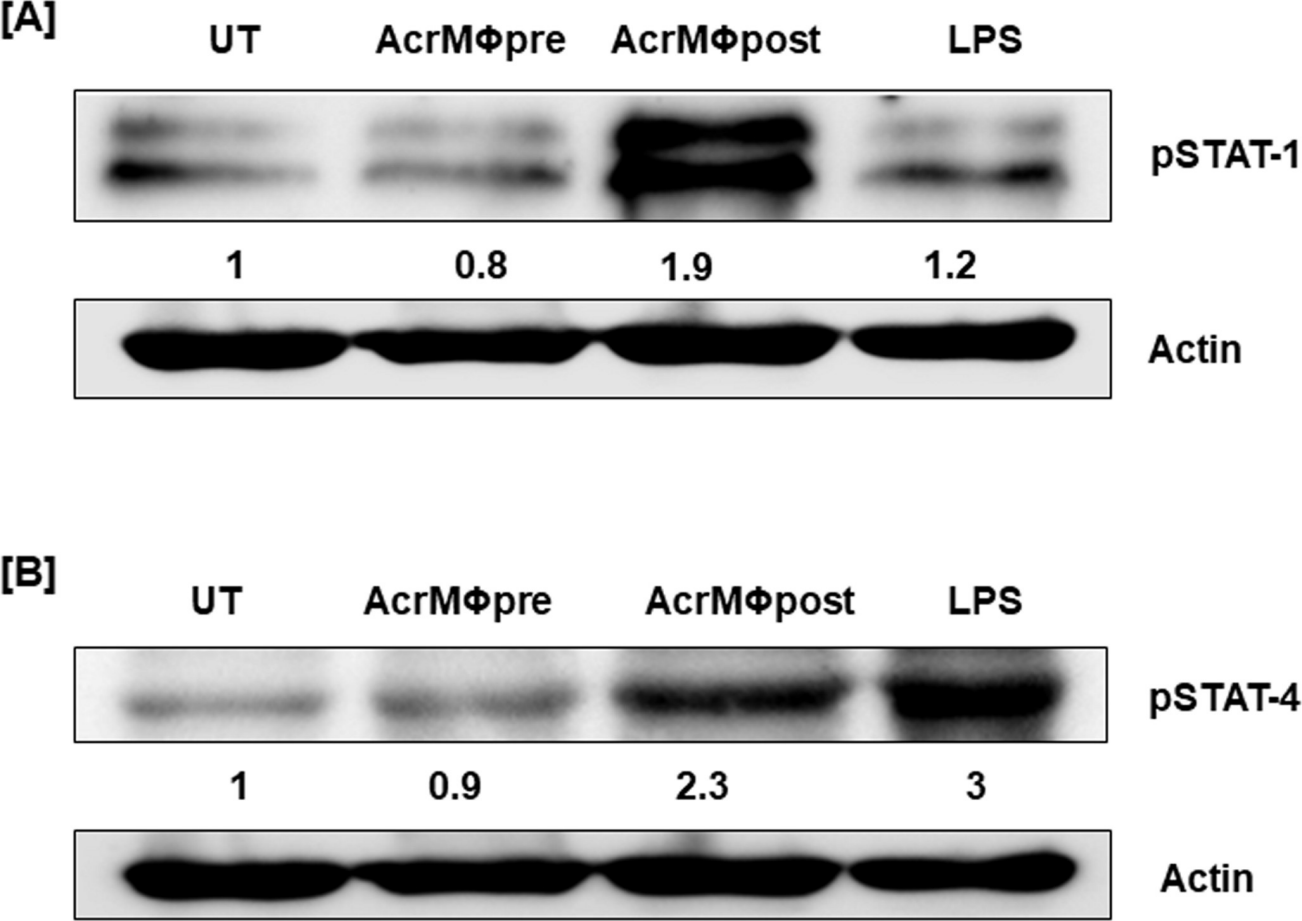
Acr1 modulates the phosphorylation of STAT molecules. Macrophages were cultured with Acr-1 (9μg/ml) during (AcrMΦpre) and after differentiation (AcrMΦpost). The cells were treated by Acr-1 (9μg/mL) for 7 days exposure of culture medium supplemented in a pre-and post-strategy. Later, the cytosolic cell lysate was made and Western blotting was completed to demonstrate the expression of **[A]** pSTAT-1; **[B]** pSTAT-4. β-Actin was taken as a loading control. The densitometry data represent fold change in expression. The density of untreated cells was considered as 1. LPS is used as positive control. The data are representative of the 2 independent experiments.

Keeping this fact into consideration, we have tried to elucidate operative mechanism conscientious for the distinct behavior of Acr-1 in both AcrMΦpre and AcrMΦpost groups. There was substantial enhancement in the expression of both STAT-1 (1.9 fold) and STAT-4 (2.3-fold) phosphorylation in AcrMΦpost group. On the other hand, expression of both STAT-1 and STAT-4 was observed in AcrMΦpre treated group **(Fig 5A and 5B)**. The extent of both STAT-1 and STAT-4 phosphorylation in AcrMΦpre treated group was considerably less as compared to unstimulated BMDM cells and AcrMΦpost treated groups. The exposure of AcrMΦpre with Acr-1 led to down regulated expression of both pSTAT-1 as well as pSTAT-4. The observed Acr-1 protein mediated decrement in STAT-1 and STAT-4 was specific and led to defective antimicrobial immunity. On the contrary, Acr-1 treatment of mature MΦs resulted in several folds increased expression of pSTAT-1 as compared to LPS. The Acr-1 treatment was also successful in upregulation of pSTAT-4 protein (2.3< fold change) in treated AcrMΦpost.

Overall, the data suggest indispensable role of pre and post treated Acr-1 in influencing the activation, maturation and differentiation of bone marrow derived macrophages (BMDM). The BMDM exhibited tolerogenic phenotype with the hampered immune response if exposed to Acr-1 during their maturation by modulating STAT-1 and STAT-4 pathways.

## Discussion

During the course of its establishment, *M*.*tb* coevolved various evading strategies to cope up hostile immune onslaught that may be waged by the host. For example, it halts fusion of phago-lysosome as a strategy to evade endo-lysosomal degradation [7, 38]. Subsequent to infection, *M*.*tb* generally switched to the dormant state and survives for years before resuscitation [39]. *M*.*tb* circumvents the immune system and endows its persistence in the host by releasing factors that modulate expression of various genes [40, 41].

The latently infected human subjects are constantly possible threat for dispersion of the disease. The invading bacterium modulates the activity of the host MΦs to withstand hostile ambiance. During hypoxic condition, many secreted *Mycobacterial* proteins modulate the host’s MΦs immune response. Many latency-associated proteins are augmented when *M*.*tb* encounters nutrient starvation, hypoxia condition, low nitric oxide or low pH that essentially mimics the granuloma environment in the lung during latent infection [42].

It has been found that *Mycobacterium* heat shock protein Acr-1 based vaccine can activate the host immune responses that protect host from subsequent *Mycobacterium* onslaught [43]. The prophylactic role of Acr-1 has been attributed to its potential to modulate MΦs and also to its capacity to trigger *M*.*tb* specific T cells in the host. Acr-1 is principally expressed during the dormant stage of *M*.*tb*. Conversely, this seems to be quite unlikely that organism which is opting dormancy as a strategy to avoid host immune system would be expressing a protein product that will act against its own survival. The present study is an attempt to solve this puzzle and can be considered as an effort to understand the possible role of Acr-1 in the modulation of host immune response.

We authenticated the role of Acr-1 in influencing the maturation and differentiation of host macrophages. Next, we explored the effect of exposure of Acr-1 to fully differentiated and mature host MΦs. The data suggest that Acr-1 can affect host immune response (especially MΦs) in above-specified condition in altogether different manners.

Although, it is well recognized that pathogen-specific immunity is governed by pathogen itself, as well as various protein expressed subsequently by pathogen at the infection site. We attempted to provide unequivocal evidence in the context of the action of *M*.*tb* specific Acr-1 protein in this regard. Novel aspects of the present study suggest; (i) the importance of T cell response in both AcrMΦpre and AcrMΦpost groups differ significantly; (ii) there is a momentous functional diversity in generated T cell response which is demonstrated in context of antigen uptake capacity of Acr-1 treated MΦs group; (iii) AcrMΦpre treatment augments an enhanced latency antigen (Acr-1) specific regulatory IL10, TIM-3, while interaction of mature MΦs with Acr-1 [AcrMΦpost] induces a specific Th17 and IFN-γ response that skewed to an inflammatory phenotype **(Fig 4)**; (iv) functional composition of Acr-1 induced cytokines in both AcrMΦpre and AcrMΦpost treated group are remarkably different. The optimal activation of naive T cells involved the interaction of the TCR by the peptide-MHC complex co-presented on the surface of macrophages. The interaction is substantially supported by costimulatory signals and also by immunoregulatory cytokines such as IL-12 [44].

Next, we explored the effect of Acr-1 on MΦs *viz*. Acr-1 modulates the activity of developing macrophages as evidenced by the exposure of MΦs with Acr-1during their maturation (AcrMΦpre) was found to result in:

- Diminish percentage of F4/80/CD86^+^cells upon treatment with AcrMΦpre.
- Downregulation of costimulatory molecules namely CD80, CD86, CD40, and MHC-II on MΦs in AcrMΦpre group.
- Decrease expression of IL-12, IL-6, and TNF-α while upregulated expression of IL-10 in AcrMΦpre group.
- Low antigen uptake ability of MΦs that offers reduced help to T cells in AcrMΦpre group.
- Induction of the tolerogenic phenotype (TIM-3^hi^) in AcrMΦpre group.
- Down regulation of STAT-1 and STAT-4 in AcrMΦpre group.
- Augmented expression of IFN-γ and IL-17 cytokine with potent allogenic T cell response in AcrMΦpost group.

We evaluated, expression of cell surface markers on Acr-1 exposed MΦs cells. Interestingly, there was significant down regulation of MHC-II, CD80, CD86, and CD40 surface molecule in AcrMΦpre group in contrast, upregulated expression of costimulatory molecules in AcrMΦpost treated group was observed in both mouse and human macrophages. Macrophages in AcrMΦpre group had phenotypically comparable cell surface expression (CD80^lo^/CD86^lo^/CD40^lo^/MHC-II^lo^/TIM-3^hi^) to the one present in chronic stages of tuberculosis granulomas [45]. Macrophages with above specified signature phenotype generally induce immune tolerance and thereby protect invading *Mycobacteria* from host immune onslaught. AcrMΦpre mediated augmentation of TIM-3 can also be correlated with the generation of tolerance [46].

In contrast, the up-regulation of the co-stimulatory surface markers in AcrMΦpost highlights the induction of mature MΦs with pre host immune response. Next, we explored the effect of Acr-1 on the expression of various cytokines in MΦs. AcrMΦpost treatment resulted in augmentation of pro-inflammatory cytokines (IL-12 and IL-6) to a significant level. TNF-α, a critical pro-inflammatory cytokine is an imperative early incident that leads to granuloma formation and help in providing protective host immune responses. We observed that Acr-1 derived AcrMΦpost enhance the production of TNF-α while there was a down regulating trend in TNF-α, expression in AcrMΦpre cells. In addition, the suppression of IFN-γ production feebly correlated with an increased level of IL-10. Acr-1 exposure of MΦs (AcrMΦpre) coincided with the significant rise in IL-10 production in agreement with the possibility of cross-regulation of IL-10 and IFN-γ. Furthermore, increased IL-10 production down regulates expression profile of the IL-12 in AcrMΦpre. Altogether, the data propose that the Acr-1 protein hampers macrophages functions in AcrMΦpre and eventually suppress Th1 type immune response in the host during *M*.*tb* infection. Acr-1 mediated debilitated functioning of macrophages ensues in less proliferation of T cells. Lower T cell function might also be linked by the CD86^lo^/TIM-3^hi^ phenotype of AcrMΦpre, which had been widely described in chronic stage diseases [47]. Acr-1 mediated activation of transcription factor STATs in bone marrow derived macrophages has direct impact on the subsequent expression of type I cytokines in the host. The present work, elucidates factors involved in cytokine signalling and regulation of immune response by latency associated protein Acr-1 of *M*.*tb* with host MΦs. Although the role of STAT-1 in *M*.*tb* infection in mice is well established [48]. STAT-1 is considered to be an important prerequisite for the maturation and activation of dendritic cell and macrophages [46]. Subsequent activation of macrophages leads to the initiation of T cells, while IL-12 secretion induces STAT-4 activation [46]. Acr-1 mediated activation of STATs has direct impact on activation of T lymphocytes in the host. It is interesting to note that Acr-1 mediated activation of macrophages (Acr-MΦpost) may induce high expression of IFN-γ that in turn causes further activation of MΦs. Employing an IFN-γ/IL-12 cytokine milieu response in pre-and post-treated Acr-MΦ cells; we observed an increase in the phosphorylation of STAT-1 and STAT-4 in AcrMΦpost group. The observed expression of STAT-1 in both the treated group (AcrMΦpre and AcrMΦpost) clearly suggest that AcrMΦpost successfully prompt MΦs for their active involvement in subsequent immune response. On the other hand, the AcrMΦpre conditions caused down regulation of both STAT-1 and STAT-4 in the treated cells.

The observation can have a strong implication in terms of high expression of IL-17 and IFN-γ in allogenic T cell response. Various operative pathways are likely to be modulated upon exposure to Acr-1. Furthermore, the establishment of factors determined by *M*.*tb* that control the JAK/STAT pathway has created up new windows to generate new anti-tubercular drug and wisely attenuated new generation vaccine, predominantly for pulmonary TB.

The present study also suggests that Acr-1 treatment during early maturation stage impairs the functioning of macrophages. It seems latency associated antigen Acr-1 play a role in the immune-evasion and immunosuppression by differential modulation of MΦs maturation and enforces them not to activate T cells for protective immunity against Mycobacterial onslaught.

Although, the present study does not provide direct evidence about the effect of Acr-1 in AcrMΦpost, it could be argued that Acr-1 could be an important antigen target for the stable design in *M*.*tb* vaccine. The vaccine approach meets the following measures: (1) availability to APCs by triggering MΦs; (2) initial recognition by the immune system in host MΦs; (3) finally initiation of Th1 response preferred proper functioning of the immune response against *M*.*tb*. Acr-1 based prophylactic scheme may need further assessment as a defensive antigen for designing new TB vaccine against active and latent TB.

## Conclusion

The current study examined *M*.*tb* dormancy associated Acr-1 protein mediated modulation of macrophage differentiation incurred through the pre and post maturational stages. The data suggest that Acr-1 affects activation, maturation, and differentiation of mouse bone marrow derived macrophages (BMDM) as well as human THP-1 monocyte derived macrophages. Exposure with Acr1 during the maturation stage results in downregulation of costimulatory molecules namely CD80, CD86, CD40, and MHC-II on MΦs in AcrMΦpre group. Further, the interaction of Acr1 with AcrMΦpre group ensues in decreased expression of IL-12, IL-6, and TNF-α on one hand and upregulated expression of IL-10 on the other. The flow cytometry and cytokine stimulation data reveal the overall increase in expression of costimulatory molecules and Th1 response in AcrMΦpost treatment groups. The BMDM exhibited tolerogenic phenotype with hampered immune response if exposed to Acr-1 during their maturation by modulating STAT-1 and STAT-4 pathways. Future studies to understand allogenic response in host macrophages may help improve functionality and specificity for increased efficacy of Acr-1 against *M*.*tb* infection.

## Supporting information

S1 Fig Acr-1 protein of *M*.*tb* augments the function of human monocytes. Human monocyte cell line (THP-1) were treated with Acr-1 (9μg/ml) during (AcrMΦpre) and after differentiation (AcrMΦpost) of monocytes to macrophages for 24h. **(A)** Untreated and Acr-1 treated monocyte were taken as cell control. **(B)** Later, the macrophages were incubated in presence or absence of Acr-1 and the expression of CD40 was monitored by flow cytometry. CFP-10 was used to establish the specificity of Acr-1. Data presented are representative of 2 independent experiments. AcrMΦpre: Acr-1 treatment during differentiation of THP-1 macrophages; AcrMΦpost: Acr-1 treatment after differentiation of THP-1 macrophages. CFP-10pre: CFP-10 treatment during differentiation of THP-1 macrophages; CFP-10post: CFP-10 treatment after differentiation of THP-1 macrophages.
(TIF)
