# Supplementary figures and images for: Mycobacterium tuberculosis host cell interaction: Role of latency associated protein Acr-1 in differential modulation of macrophages
Source: PLoS One. 2018 Nov 5;13(11):e0206459. doi: 10.1371/journal.pone.0206459 (PMC6218195; doi:10.1371/journal.pone.0206459)

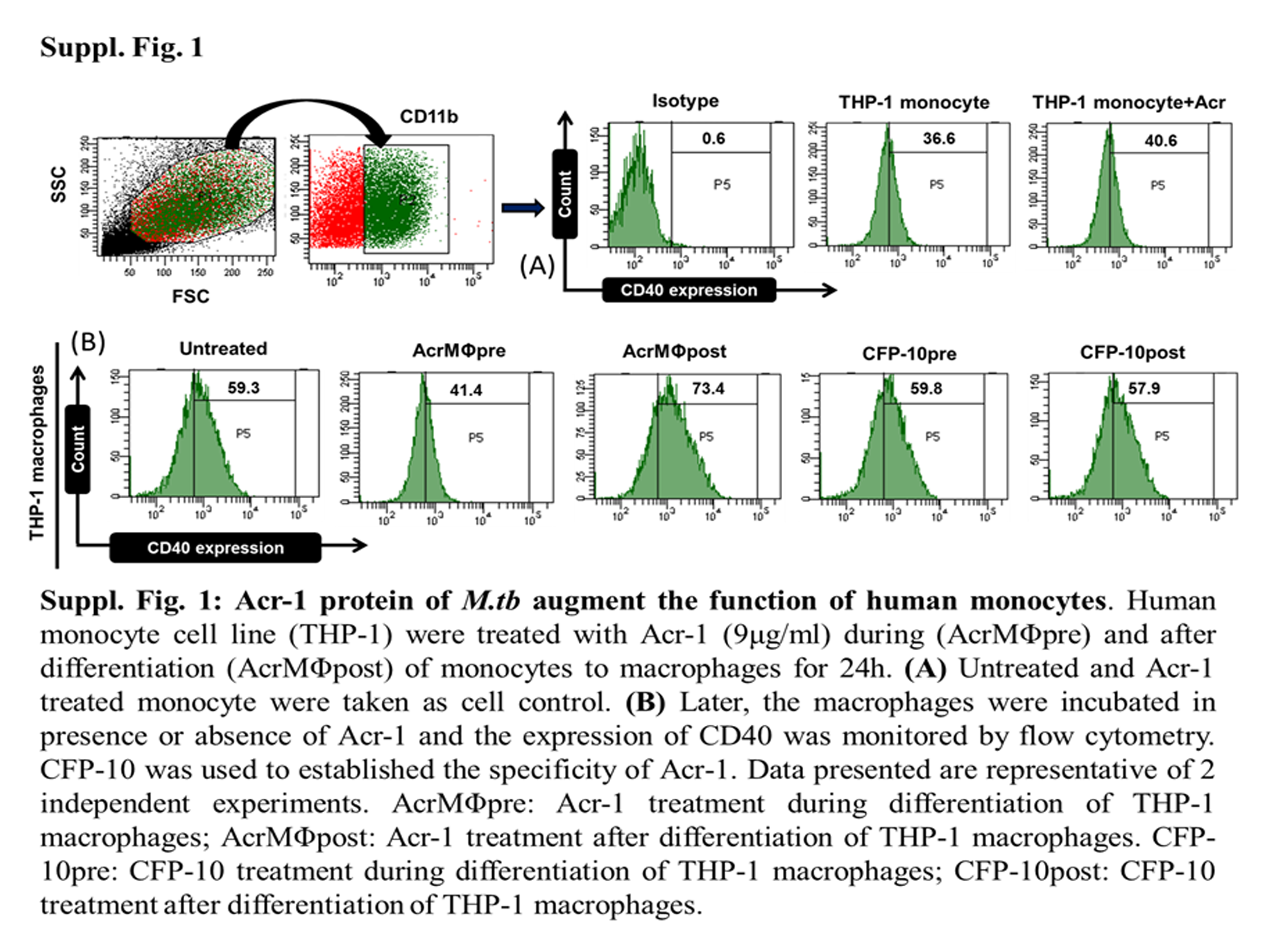

Supplement: S1 Fig — Human monocyte cell line (THP-1) were treated with Acr-1 (9μg/ml) during (AcrMΦpre) and after differentiation (AcrMΦpost) of monocytes to macrophages for 24h. (A) Untreated and Acr-1 treated monocyte were taken as cell control. (B) Later, the macrophages were incubated in presence or absence of Acr-1 and the expression of CD40 was monitored by flow cytometry. CFP-10 was used to establish the specificity of Acr-1. Data presented are representative of 2 independent experiments. AcrMΦpre: Acr-1 treatment during differentiation of THP-1 macrophages; AcrMΦpost: Acr-1 treatment after differentiation of THP-1 macrophages. CFP-10pre: CFP-10 treatment during differentiation of THP-1 macrophages; CFP-10post: CFP-10 treatment after differentiation of THP-1 macrophages. (TIF) [file pone.0206459.s001.tif]
